# Effects of bisphenol-S low concentrations on oxidative stress status and *in vitro* fertilization potential in mature female mice

**Published:** 2017-12-15

**Authors:** Alireza Nourian, Ali Soleimanzadeh, Ali Shalizar Jalali, Gholamreza Najafi

**Affiliations:** 1 *Department of Theriogenology, Faculty of Veterinary Medicine, Urmia University, Urmia, Iran;*; 2 *Department of Basic Sciences, Faculty of Veterinary Medicine, Urmia University, Urmia, Iran.*

**Keywords:** Bisphenol-S, *In vitro* fertilization, Mouse, Oxidative stress

## Abstract

Bisphenol-S (BPS) is a new bisphenol-A substitute widely used in many plastic products. Bisphenol-A as a main member of bisphenol family has been known as an endocrine system disrupter chemical compound. Like other members of bisphenol family, there is public health concern about the toxic effects of BPS on reproductive system, thus, we examined BPS effects on *in vitro* fertilization (IVF) potential and oxidative stress status in a murine model. Adult female mice (n = 70) were randomly divided into control and BPS-treated groups. Bisphenol-S was administered at doses of 0, 1, 5, 10, 50 and 100 µg kg^-1 ^body weight per day intraperitoneally for 21 consecutive days. Twenty-Four hr after the last treatment, five mice in each group were super-ovulated and the oocytes were harvested for IVF. All ovaries were collected and used for biochemical factors analyses. Bisphenol-S exposure at doses more than 10 µg kg^-1 ^induced developmental arrest of pre-implantation embryos. Further, lipid peroxidation measurement in ovaries indicated that all doses of BPS cause oxidative stress in female mice. In conclusion, BPS administration even in low doses can result in female reproductive toxicities and oxidative stress in mice.

## Introduction

Endocrine disrupter chemicals (EDCs) are exogenous chemicals which interrupt the physiological function of estrogen due to their high affinity to estrogen receptors.^1^ Because of the harmful potential of EDCs on human health, they have been widely investigated in the last two decades.^2^ Bisphenol-A (BPA) is one of the main EDCs being used in many modern life materials such as plastic containers, adhesives, paints, dental sealants, infants feeding bottles and paper products.^3^ The worldwide production of BPA was nearly over three million tons in 2003.^4^ The BPA can be absorbed through ingestion, inhalation and skin and can be detected in blood, urine, amniotic fluid and fetal plasma samples in nMol concentration which is harmful for fetal development and differentiation.^5^

Previously, detrimental effects of BPA on female reproductive system have been shown.^6^^-^^11^ The BPA has been detected in serum and follicular fluid of women with polycystic ovary syndrome.^12^^,^^13^ Moreover, *in* *utero* exposure of BPA affects early ovarian development in pregnant mice and inhibits germ cell nest breakdown in ovary.^14^

Because of public health concern, many countries use BPA-substitutes in their new products, however, toxic effects of these materials have been addressed recently in animal model studies.^15^

Bisphenol-S (BPS), a BPA-substitute, is widely used in many new products such as baby bottles and food containers, especially BPA-free labeled ones and its production increases annually.^15^^,^^16^ The BPS was named as a safe substitute because of better stability at high temperature and sunlight and lesser estrogenic activity.^17^^-^^21^

Estrogenic effects as well as oxidative properties of bisphenol family were examined previously. It has been reported that they can induce oxidative stress in many tissues including testis, brain, liver and kidney.^22^^,^^23^ It was found that BPS has less oxidative effects on blood cells compared to BPA, bisphenol-F (BPF) and bisphenol-AF, but BPS and BPA have much destructive effects on proteins.^16^^,^^24^ *In vitro* study on human peripheral blood mononuclear cells revealed that BPS doesn’t induce significant DNA damage.^25^

Accordingly, administration of BPA induced dose dependent testicular toxicity in mice and rats.^26^ Further, it has been reported that BPA reduces sperm quality via disruption of extracellular-signal-regulated kinase pathway.^27^ Recently, evaluation of BPS and BPF effects on human and mouse fetal testis cultures showed that BPS can suppress testosterone production.^28^ Histological and biochemical evidence in rat model study demonstrated that BPS causes testicular toxicities.^29^

Based on this concept, current study was designed to elucidate the dose dependent effects of BPS exposure on *in vitro* fertilization (IVF) outcome and oxidative stress status by using mice as an animal model.

## Materials and Methods

**Chemicals. **The BPS (99%, 4, 4′-Sulfonyldiphenol), (CAS No. 80-09-1) and ethanol (ACS grade; CAS No. 64-17-5) were purchased from Sigma-Aldrich Company (St. Louis, USA). 

**Animals. **Seventy sexually adult, same cycling stage, female mice (age 70-80 days) were obtained from Animal Resources Center of the Faculty of Veterinary Medicine, Urmia University. In the first stage of study, estrus cycle stage was determined by vaginal smears for all mice.^30^ Mice were housed in groups of ten animals per case (standard cage) with 12-12 hr dark-light cycle. Animals were fed by soy free food and had free access to water. The experiments were performed on animals in accordance with the guidelines of the ethical committee for research on laboratory animals of Urmia University (3/PD/47, 2016).

**Experimental protocol. **Mice were adapted to environment for seven days. Different doses (0, 1.00, 5.00, 10.00, 50.00 and 100.00 µg kg^-1 ^body weight per day; IP) of BPS were used for 21 consecutive days in this study based on previous studies and one group was served as a control group.^31^ Twenty-four hr after the last treatment, five mice in each group were euthanized by combination of ketamine (45 mg kg^-1^; IP) and xylazine (35 mg kg^-1^; IP).^31^ Then, ovaries were collected immediately and used for biochemical analyses. 

**Oocytes collection and IVF assays. **Five mature female mice were chosen randomly in each group and super-ovulated as previously described.^32^ Five to 10 IU of pregnant mare’s serum gonadotropin (PMSG; Intervet International BV, Boxmeer, The Netherlands) and 7.50 IU human chorionic gonadotropin (hCG; Intervet International BV) were injected intraperitoneally 48 hr and 12 hr before experiment into all mice, respectively. Human tubal fluid (HTF; Sigma) medium was equilibrated with 5% CO_2_ at 37 ^°^C in incubator (SINA Company, Tehran, Iran) for 24 hr before experiment. Mouse sperm was prepared by harvesting one mouse caudal epididymis and incubated for 1 hr in HTF medium before the experiment for capacitation. In the day of experiment, all cumulus-oocyte complexes were collected and incubated for 1 hr. Metaphase II arrested mouse oocytes were inseminated with capacitated sperms as described above and 4 hr later, all fertilized zygotes were transferred to HTF cleavage medium. All zygotes were evaluated 24 hr, 48 hr and five days after insemination. Intact, fragmented and/or lysed embryos which did not develop, were recorded as arrested embryos. In the current study, the rate of cell lyses was recorded as follows: Type I: fully lysed, necrotic and/or fragmented embryos, Type II: embryos with partially lysed/fragmented blastomeres and Type III: embryos with some lysed/fragmented blastomeres and/or cytoplasmic vesicles.^33^

**Lipid peroxidation. **Malondialdehyde (MDA) is commonly used as an indicator of lipid peroxidation in oxidative stress status examination. A volume of 300 µL of 10.00% trichloro-acetic acid (Sigma) was added to150 µL of ovarian tissue samples and centrifuged at 1000 rpm for 10 min at 4 ˚C, then, incubated in 300 µL of 67.00% thio-barbituric acid (TBA; Sigma) at 100 ˚C for 25 min. Five min after cooling of the solution, pink color was appeared because of MDA-TBA reaction and evaluated using a spectrometer (model Novaspec II; Biochrom Ltd., Cambridge, UK) at a wave length of 535 nm.^34^

**Statistical analysis. **The data were analyzed by SPSS (version 20; SPSS Inc., Chicago, USA) and one-way ANOVA. A *p* value less than 0.05 was considered statistically significant.

## Results

***In vitro*** **fertilization. **As shown in Table 1, BPS exposure at 10.00, 50.00 and 100.00 µg kg^-1 ^caused significant reduction in fertilization rate. The 2-cell embryo percentage was changed even in low doses of BPS, however, doses more than 10.00 µg kg^-1 ^induced meaningful reduction. In addition, blastocyst rate reduction was statistically significant following 10.00, 50.00 and 100.00 µg kg^-1 ^BPS administration. Moreover, 5.00 µg kg^-1 ^of BPS induced reduction in blastocyst rate which wasn’t statistically meaningful. According to Table 2, all concentrations of BPS inhibited embryo development and increased embryo arrest, however, statistical analyses revealed that BPS treatment only at doses of 50.00 and 100.00 µg kg^-1 ^resulted in significant increase of total and type I embryo arrests.

**Lipid peroxidation. **The antioxidant status in ovarian tissues based on MDA measurement in control, control sham, BPS 1, BPS 5, BPS 10, BPS 50 and BPS 100, groups were 3.87 ± 0.94, 3.80 ± 0.83, 4.52 ± 1.13, 4.97 ± 1.07, 6.26 ± 0.71, 8.73 ± 1.39 and 9.11 ± 1.23, respectively. The BPS administration led to MDA elevation in mice ovaries in a dose dependent manner. 

**Table 1:** Dose dependent effect of bisphenol-S on *in vitro* fertilization outcome in experimental groups. Data are presented as mean ± SE.

**Groups**	**Oocyte number**	**Fertilization rate (%)**	**2-cell embryos (%)**	**Blastocysts (%)**
**Control**	88	80.68 ± 1.18(71.00)^a^	85.91 ± 0.83(61.00) ^a^	59.15 ± 1.40(42.00) ^a^
**Control sham**	86	83.72 ± 0.79(72.00) ^a^	86.11 ± 1.45(62.00) ^a^	59.72 ± 1.12(43.00) ^a^
**BPS 1**	82	80.48 ± 1.21(66.00) ^a^	83.33 ± 1.35(55.00)^ac^	57.57 ± 1.45(38.00) ^a^
**BPS 5**	75	77.33 ± 1.30(58.00) ^a^	77.58 ± 0.69(45.00) ^ac^	56.89 ± 0.84(33.00)^ab^
**BPS 10**	61	60.65 ± 0.92(37.00)^b^	67.56 ± 0.81(25.00) ^ac^	51.35 ± 0.79(19.00) ^b^
**BPS 50**	57	50.87 ± 1.16(29.00) ^b^	65.51 ± 1.47(19.00) ^ac^	31.03 ± 1.31(9.00)^c^
**BPS 100**	37	56.75 ± 0.63(21.00) ^b^	61.90 ± 1.15(13.00) ^ac^	33.33 ± 0.94(7.00)^c^

abc Means within a column with different superscript letters denote significant differences (*p* < 0.05).

**Table 2:** Dose dependent effect of bisphenol-S on embryo arrest in experimental groups. Data are presented as mean ± SE.

**Groups**	**Arrested embryos (%)**	**Type 1 (%)**	**Type 2 (%)**	**Type 3 (%)**
**Control**	40.84 ± 0.48(29.00) ^a^	7.40 ± 0.83(5.00)^a^	9.85 ± 1.19(7.00) ^a^	23.94 ± 0.72(17.00) ^a^
**Control sham**	40.27 ± 1.16(29.00.00) ^a^	2.77 ± 0.51(2.00) ^a^	12.50 ± 0.65(9.00)^ac^	25.00 ± 1.18(18.00) ^a^
**BPS 1**	42.42 ± 0.76(28.00) ^a^	9.09 ± 0.89(6.00) ^ac^	15.15 ± 0.92(10.00) ^ac^	18.18 ± 1.32(12.00) ^a^
**BPS 5**	43.10 ± 0.39(25.00)^ac^	10.34 ± 0.47(6.00)^ad^	13.79 ± 1.26(8.00) ^ac^	18.96 ± 0.49(11.00) ^a^
**BPS 10**	48.64 ± 1.24(18.00)^ab^	21.62 ± 0.71(8.00)^bcd^	13.51 ± 1.40(5.00) ^ac^	13.51 ± 0.81(5.00) ^a^
**BPS 50**	68.96 ± 1.49(20.00)^bd^	34.48 ± 0.63(10.00)^b^	24.13 ± 0.79(7.0)^bc^	10.34 ± 1.64(3.00) ^a^
**BPS 100**	66.66 ± 0.55(14.00)^bcd^	28.57 ± 1.24(6.00)^bcd^	28.57 ± 0.97(6.00) ^ac^	9.52 ± 1.04(2.00) ^a^

abcd Means within a column with different superscript letters denote significant differences (*p* < 0.05).

## Discussion

The BPS, a substitute of BPA, has been introduced to the manufacture as a safer alternative to BPA in multiple products.^35^ Moreover, BPS exhibits less estrogenic activities in comparison to BPA.^36^ Similarly, BPS has been shown to have apoptotic and oxidative properties as well as toxic effects in male reproductive system and it induces DNA damages.^16^^,^^29^^,^^37^^-^^39^ This study was conducted to determine the effects of BPS on female mice fertility and oxidative stress status in ovarian tissue. 

Recently, EDCs effects on fertility have caused great concern.^40^^,^^41^ In our study, fertilization rates, 2-cell embryo numbers and blastocyst percentages reduced significantly in 10.00, 50.00 and 100.00 µg kg^-1 ^groups compared to control group. Comparison between higher (10.00, 20.00 and 50.00 µg kg^-1^) and lower (0.00, 1.00 and 5.00 µg kg^-1^) doses of BPS, revealed that BPS doesn’t have significant effects on IVF outcomes in low doses. However, there were slight reductions in 2-cell and blastocyst rates in lower dosages of BPS which were not statistically meaningful. Previous studies have reported that BPS causes male and female gonadal weight along with egg production and hatchability reductions.^15^ Furthermore, it has been suggested that BPA concentration in blood and follicular fluid can be associated with embryo quality, implantation rate and IVF outcomes.^42^ It has been shown that low (1.00 µg L^-1^) and high (100.00 µg L^-1^) concentrations of BPS have negative impacts on egg production, fertilization and hatching rates which confirm our findings.^43^

In this study, higher doses of BPS induced remarkable oxidant/antioxidant imbalance in ovarian tissue compared to lower doses, however, all BPS-treated groups showed MDA elevation in ovarian tissues compared to the control group. These findings are in agreement with the previous report in which it was indicated that BPS disturbs oxidant/antioxidant balance in testicular tissue.^29^ Furthermore, *in vitro* studies have demonstrated that BPS plays important roles in oxidative stress induction.^25^^,^^41^^,^^44^^,^^45^

In conclusion, this study highlighted hidden aspects of BPS exposure particularly in female reproductive system and early embryo development. Further studies are required to uncover precise mechanisms of BPS-induced embryo toxicities.
